# Balanced Diet-Fed Fat-1 Transgenic Mice Exhibit Lower Hindlimb Suspension-Induced Soleus Muscle Atrophy

**DOI:** 10.3390/nu9101100

**Published:** 2017-10-06

**Authors:** Gabriel Nasri Marzuca-Nassr, Gilson Masahiro Murata, Amanda Roque Martins, Kaio Fernando Vitzel, Amanda Rabello Crisma, Rosângela Pavan Torres, Jorge Mancini-Filho, Jing Xuan Kang, Rui Curi

**Affiliations:** 1 Department of Internal Medicine, Faculty of Medicine, Universidad de La Frontera, Temuco 4780000, Chile; 2 Department of Physiology and Biophysics, Institute of Biomedical Sciences, University of São Paulo, São Paulo 05508-000, Brazil; gil_masa@yahoo.com.br (G.M.M.); amandarmartins.arm@gmail.com (A.R.M.); vitzel@gmail.com (K.F.V.); amycrisma@yahoo.com.br (A.R.C.); ruicuri59@gmail.com (R.C.); 3 School of Health Sciences, College of Health, Massey University, Auckland 0632, New Zealand; 4 Laboratory of Lipids, Department of Food Science and Nutrition, Faculty of Pharmaceutical Sciences, University of São Paulo, São Paulo 05508-000, Brazil; rptorres@usp.br (R.P.T.); jmancini@usp.br (J.M.-F.); 5 Laboratory for Lipid Medicine and Technology, Massachusetts General Hospital and Harvard Medical School, Boston, MA 02114, USA; kang.jing@mgh.harvard.edu; 6 Interdisciplinary Post-Graduate Program in Health Sciences, Cruzeiro do Sul University, São Paulo 05508-000, Brazil

**Keywords:** Fat-1 mice, muscle disuse atrophy, ω-3 PUFAs, hindlimb suspension, protein synthesis/degradation signaling

## Abstract

The consequences of two-week hindlimb suspension (HS) on skeletal muscle atrophy were investigated in balanced diet-fed Fat-1 transgenic and C57BL/6 wild-type mice. Body composition and gastrocnemius fatty acid composition were measured. Skeletal muscle force, cross-sectional area (CSA), and signaling pathways associated with protein synthesis (protein kinase B, Akt; ribosomal protein S6, S6, eukaryotic translation initiation factor 4E-binding protein 1, 4EBP1; glycogen synthase kinase3-beta, GSK3-beta; and extracellular-signal-regulated kinases 1/2, ERK 1/2) and protein degradation (atrophy gene-1/muscle atrophy F-box, atrogin-1/MAFbx and muscle RING finger 1, MuRF1) were evaluated in the soleus muscle. HS decreased soleus muscle wet and dry weights (by 43% and 26%, respectively), muscle isotonic and tetanic force (by 29% and 18%, respectively), CSA of the soleus muscle (by 36%), and soleus muscle fibers (by 45%). Fat-1 transgenic mice had a decrease in the ω-6/ω-3 polyunsaturated fatty acids (PUFAs) ratio as compared with C57BL/6 wild-type mice (56%, *p <* 0.001). Fat-1 mice had lower soleus muscle dry mass loss (by 10%) and preserved absolute isotonic force (by 17%) and CSA of the soleus muscle (by 28%) after HS as compared with C57BL/6 wild-type mice. p-GSK3B/GSK3B ratio was increased (by 70%) and MuRF-1 content decreased (by 50%) in the soleus muscle of Fat-1 mice after HS. Balanced diet-fed Fat-1 mice are able to preserve in part the soleus muscle mass, absolute isotonic force and CSA of the soleus muscle in a disuse condition.

## 1. Introduction

Skeletal muscle disuse-induced atrophy is associated with decreases in skeletal muscle mass, cross-sectional area, contractile force, and protein synthesis signaling activity [1,2,3,4]. Physical exercise [5,6,7,8,9], neuromuscular electrical stimulation [10,11], and dietary supplementation [12,13,14,15] are employed to attenuate skeletal muscle loss in patients and animal experimental models. ω-3 Polyunsaturated fatty acids (PUFAs)-rich fish oil increases skeletal muscle function in elderly patients [16,17] and attenuates skeletal muscle mass decrease induced by muscle immobilization [18] and cancer cachexia [19] in rodents. ω-3 PUFAs activate protein synthesis (Protein kinase B/ mammalian target of rapamycin/p70 ribosomal protein S6 kinase, Akt/mTOR/p70S6K) [18,20,21,22,23] and inhibit protein degradation signaling pathways (atrophy gene-1/muscle atrophy F-box, atrogin-1/MAFbx and muscle RING finger 1, MuRF1) [18]. We recently reported [1] the effects of oral supplementation of “high dose” of either eicosapentaenoic (EPA)- or docosahexaenoic (DHA)-rich fish oil on protein synthesis/degradation signaling pathways in skeletal muscle mass loss induced by two weeks of hindlimb suspension (HS) in rats. EPA-rich fish oil attenuated the changes induced by HS on 26S proteasome activity, CSA of soleus muscle fibers, and levels of p-Akt, total p70S6K, p-p70S6K/total p70S6K, p-4EBP1 (eukaryotic translation initiation factor 4E-binding protein 1), p-GSK3-beta (glycogen synthase kinase3-beta), p-ERK (extracellular-signal-regulated kinases) 2, and total ERK 1/2 proteins. DHA-rich fish oil attenuated the changes induced by HS on p-4EBP1 and total ERK1 levels [1].

Jiang X. Kang developed, in 2004, the Fat-1 transgenic mice that produce ω-3 PUFAs from dietary ω-6 PUFAs [24]. Experimental models of spinal cord injury [25] and peripheral nerve injury [26] were investigated in Fat-1 transgenic mice fed with a ω-6 PUFA-rich diet. The strain displayed neuroprotective properties and the authors reported attenuation of skeletal muscle atrophy due to peripheral nerve injury [26]. Whether the primary cause was an effect in the nervous system or in skeletal muscle tissue itself was not addressed. Also, the loss of muscle mass in Fat-1 mice was not yet evaluated using muscle atrophy experimental models that maintain neural activity.

The consequences of HS-induced muscle atrophy in balanced diet-fed Fat-1 mice were investigated. The purpose was to investigate whether small changes in the ω-6/ω-3 PUFAs ratio, in “long term”, would be able to attenuate skeletal muscle disuse-induced atrophy. For this purpose, the following measurements were performed: body weight gain, hindlimb skeletal muscle wet and dry mass, soleus muscle and soleus muscle fibers CSA and force production by the soleus muscle. Skeletal muscle signaling pathways associated with protein synthesis (Akt, S6, 4EBP1, GSK3B and ERK 1/2 content) and protein degradation (cathepsin L and 26S proteasome activities and contents of atrogin-1/MAFbx and MuRF1) were also measured. 

## 2. Materials and Methods

### 2.1. Animals

The animals were maintained at the Department of Physiology and Biophysics, Institute of Biomedical Sciences, University of São Paulo under 12 hours light/dark cycle and food (commercial diet; Nuvilab-CR1, Quimtia-Nuvital, Colombo, Brazil) and water *ad libitum*. Fat-1 transgenic heterozygous mice were cross-bread with C57BL/6 wild-type mice [24,27]. The offspring genotypes were evaluated by standard RT-PCR (real time polymerase chain reaction) at 21 day-old to confirm C57BL/6 wild-type mice or Fat-1 transgenic mice lineage (Figure S1A). Ethics Committee of the Institute of Biomedical Sciences, University of São Paulo, approved the experimental protocols used in this study (CEUA.138/2013). 

### 2.2. Experimental Design

Eight-week-old mice were divided into four groups: C57BL/6 wild-type (C57BL/6, *n* = 20), C57BL/6 wildtype submitted to hindlimb suspension (C57BL/6 + HS, *n* = 18), Fat-1 (Fat-1, *n* = 15), and Fat-1 submitted to hindlimb suspension (Fat-1 + HS, *n* = 15). Total number of animals in the different groups was in 3 separated sets of experiments. The animals were left to adapt to individual cages for 3 days. Afterwards (at Day 0), the suspension of the hind limb was initiated and maintained for two weeks. After this period, the mice were anesthetized by intraperitoneal injection of ketamine (90 mg/kg b.w.) and xylazine (10 mg/kg b.w.), weighed and killed by exsanguination. Muscles (soleus, gastrocnemius, plantaris, tibialis anterior and extensor digitorum longus (EDL)) of both limbs and fat depots (subcutaneous, epididymal, retroperitoneal, and mesenteric) were removed, weighed and stored at −80 °C.

#### Hindlimb Suspension (HS)

Same HS experimental protocol has been reported in studies by others [28,29] and in our previous study in rats [1]. In short, the rats were maintained in individual special cages with the tail attached on the top of the cage, using a tape. In this position, the hind limbs of the animals cannot contact the floor, removing the mechanical loading and causing skeletal muscle disuse atrophy in the hind limbs. 

### 2.3. Gastrocnemius Fatty Acid Composition 

Fatty acid composition was determined in the gastrocnemius muscle (~30 mg of fresh tissue) by gas chromatography using the AOAC 996.06 [30] and AOCS Ce 1j-07 [31] methods as described in our previous studies [1,32]. The composition of fatty acids in the gastrocnemius muscle was expressed as g/100 g tissue wet weight.

### 2.4. In Vivo Soleus Muscle Electrical Stimulation

The experimental protocol used was based on Wojtaszewski et al. [33] and Silveira et al. [34] with modifications by Pinheiro et al. [35], Fortes et al. [36], and Abreu et al. [32]. This protocol was the same described in our previous studies in rats [32,35,36]. The animals were anesthetized as described above and fixed on an acrylic platform. The stimulated hind limb was fitted on the platform with the hip and knee joints fixed at ~130° angle. A hook was placed under the Achilles tendon for measurement of the contractile properties of the soleus muscle using a force transducer (Grass Technologies, West Warwick, RI, USA), after tenotomy of synergistic and antagonist muscles. Electrical stimulation device MultiStim System D330 (Digitimer Ltd, Welwyn Garden City, Hertfordshire, UK) connected to an electrode placed at the sciatic nerve was used to promote soleus muscle contractions. The resting length (L0) of the soleus muscle was adjusted to produce maximum tension upon stimulation (obtained with the ankle joint at approximately 90° angle). 

### 2.5. Analysis of Soleus Muscle Force and Contractile Properties 

The procedures used were the same described in our previous studies for rats [32,35,36]. The AqDados software (version 4.16, Lynx Tecnologia Eletronica Ltda, São Paulo, Brazil) was used to record the muscle isotonic force generated during twitches at 1 Hz frequency and 500 μs pulse duration. Five contractions were used to calculate the absolute isotonic force production, time to peak (TTP—time between the onset of force development until peak tension), half relaxation time (HRT—time of muscle relaxation half-way from peak tension), and late relaxation time (LRT—time of muscle relaxation between 50% and 25% of peak tension). The tension generated at 100 Hz and 500 μs pulse duration was used to calculate muscle tetanic force. The absolute tetanic force was determined through maximum force production obtained through voltage adjustment. The AqAnalysis software (version 4.16, Lynx Tecnologia Eletronica Ltda, São Paulo, Brazil) was used to evaluate muscle force. 

### 2.6. Analysis of Soleus Muscle Resistance to Fatigue 

The procedure used was the same described in our previous studies in rats [32,37,38]. The skeletal muscle fatigue-inducing protocol consisted of 10 tetanic contractions with 2 s duration each. The soleus muscle was stimulated every 10 s and peak muscle force was obtained on each contraction for the calculation of the area under the curve (force × contraction), named as muscle fatigue index [35].

### 2.7. Histological Analysis of the Soleus Muscle

CSA of the soleus muscle and soleus muscle fibers were calculated according to Bodine and Baar [39] and as described in our previous study [1]. The slides obtained from sections of the central portion of the soleus muscle were stained with hematoxylin and eosin (HE). Photographs were taken using an optical microscope (Nikon Eclipse E1000, Fukuoka, Japan) attached to a digital camera (Nixon DXM 1200). The images were analyzed (150 fibers per muscle) using the AxioVision program (version 4.8.1.0, Carl Zeiss Imaging Solutions, Jena, Germany). 

### 2.8. Determination of Cathepsin L Activity in the Gastrocnemius Muscle

Cathepsin L activity was determined in the gastrocnemius muscle using the Cathepsin L Activity Assay Kit (Abcam—ab65306, Abcam Inc., Cambridge, UK), according to the recommendations of the manufacturer. The method was used in our previous study [1] and described by Jannig et al. [40]. 

### 2.9. 26S Proteasome Activity Measurement in the Gastrocnemius Muscle

Gastrocnemius muscle 26S proteasome activity was determined as described by Churchill et al. [41] and Cunha et al. [42]. This assay was also used in our previous study [1]. In short, gastrocnemius muscle was homogenized in a buffer (210 mmol/L D-mannitol; 70 mmol/L sucrose; 5 mmol/L MOPS; and 1 mmol/L EDTA; pH 7.4). After centrifugation, the supernatant was used for determination of cytosolic proteins. The activity of the chymotrypsin site of the 26S portion of the proteasome was assessed by fluorometric assay. Measurements were performed in the absence and presence of epoxomicin (20 lmol/L). The difference between the two rates was attributed to the proteasome activity [42].

### 2.10. Analysis of Akt, S6, 4EBP1, GSK3-Beta, Atrogin-1/MAFbx, MuRF1 and ERK 1/2 in the Soleus Muscle by Western Blot

Soleus muscles were homogenized and the total content of proteins was determined as previously described [43]. Equal amounts of total protein (10 μg) were used to determine the content of signaling molecules involved in the protein synthesis and degradation pathways as described in our previous study [1]. Total loading of proteins for each sample, as indicated by the Ponceau S staining, was used to normalize the results (Figure S1B) [1,44,45,46], expressed in values relative to the C57BL/6 control group. A pool sample, composed of equal parts of all experimental condition samples, was used for normalization among membranes. The images were captured by the Amersham Imager 600 (Amersham/GE Healthcare) and quantified using the Image J software (NIH, Bethesda, MD, USA) (Figure S1C). The primary antibodies used (dilution 1:1000) were: p-Akt at Ser 473 (9271), Akt (9272), p-S6 (ribosomal protein S6) at Ser 240/244 (5364), S6 (2217), p-4EBP1 at Thr 37/46 (2855), 4EBP1 (9644), p-GSK3-beta at Ser 9 (9323), GSK3-beta (9315), p-ERK 1/2 at Thr 202/Tyr 204 (9101), and ERK 1/2 (4695) from Cell Signaling Technology (Danvers, MA, USA); and atrogin-1 (AP2041) and MuRF1 (MP3401) from ECM Biosciences (Versailles, KY, USA). 

### 2.11. Statistical Analysis

Statistical analysis was performed using the GraphPad Prism^®^ software (version 4.01; El Camino Real, CA, USA). Results are presented as mean ± standard error of the mean (SEM). The overall differences for C57BL/6 and C57BL/6 + HS vs. Fat-1 and Fat-1 + HS or for C57BL/6 and Fat-1 vs. C57BL/6 + HS and Fat-1 + HS (main effects for Fat-1 or HS) were analyzed by two-way analysis of variance (ANOVA). When significant difference was found, the Bonferroni *post-hoc* test was used for differences between specific groups being detected. Grubb’s test was used to exclude outliers. The differences were considered significant for *p <* 0.05.

## 3. Results

### 3.1. Body Weight Gain

The C57BL/6 group had a body weight gain of 1.6 ± 0.3 g and the Fat-1 group 1.3 ± 0.4 g over the two-week experimental period. The HS promoted a decrease in the body weight gain in both groups: −0.7 ± 0.3 for the C57BL/6 and −1.7 ± 0.8 for the Fat-1 mice (Table 1).

### 3.2. Wet and Dry Mass of the Soleus, Gastrocnemius, Plantaris, Tibialis Anterior, and EDL Muscles, and Total Protein Content in the Soleus Muscle 

HS decreased soleus muscle wet and dry mass in C57BL/6 mice by 42% and 25%, respectively, and in Fat-1 mice by 27% and 9%, respectively. As indicated by ANOVA and the Bonferroni post-test, Fat-1 mice had a less pronounced reduction of soleus muscle mass due to HS (Table 1). The gastrocnemius muscle wet and dry mass were decreased by 23% and 22%, respectively, in C57BL/6 mice and by 17% and 18%, respectively, in Fat-1 mice. Plantaris muscle wet and dry mass were reduced in C57BL/6 mice by 22%, and in Fat-1 mice by 14% and 19%, respectively, due to HS. Tibialis anterior muscle wet and dry mass were decreased in C57BL/6 mice by 17% and 11%, respectively, and in Fat-1 mice by 18% and 17%, respectively. HS reduced EDL muscle wet and dry weights in C57BL/6 mice by 9% and 14%, respectively, and in Fat-1 mice by 14% and 7%, respectively. The effects of HS on soleus muscle dry mass, gastrocnemius muscle wet and dry mass, plantaris muscle dry mass, tibialis anterior muscle wet and dry mass and EDL muscle dry weight were not different between C57BL/6 and Fat-1 mice as indicated by ANOVA and the Bonferroni *post-hoc* test. Soleus muscle total protein content (mg/g fresh tissue) was significantly higher (*p <* 0.05) in Fat-1 as compared with C57BL/6 wild-type mice (Table 1).

### 3.3. Wet Mass of Subcutaneous, Epididymal, Retroperitoneal and Mesenteric Fat Depots

HS had no significant effect on subcutaneous and retroperitoneal fat depots in C57BL/6 mice. However, HS decreased subcutaneous fat depot mass by 12% and retroperitoneal fat mass by 32% as compared with non-HS Fat-1 mice. The epididymal fat mass was reduced due to HS (*p <* 0.001); by 41% in C57BL/6 mice and by 44% in Fat-1 mice. HS decreased the mesenteric fat mass by 22% in C57BL/6 mice and by 17% in Fat-1 mice (Table 1). Fat-1 had a significant reduction in epididymal (C57BL/6 vs. Fat-1 groups, *p <* 0.01) and retroperitoneal (C57BL/6 + HS vs. Fat-1 + HS groups, *p <* 0.05) fat depots as indicated by Bonferroni *post-hoc* test. 

### 3.4. Gastrocnemius Muscle Fatty Acid Composition

As indicated by ANOVA and the Bonferroni *post-hoc* test, the ω-6/ω-3 PUFA ratio was decreased in the gastrocnemius muscle of the C57BL/6 + HS group as compared with C57BL/6 mice (*p <* 0.01) and of Fat-1 as compared with C57BL/6 mice either without HS (C57BL/6 vs. Fat-1, *p <* 0.001) or with HS (C57BL/6 + HS vs. Fat-1 + HS, *p <* 0.01). The values were: 2.62 ± 0.39 for C57BL/6; 2.11 ± 0.27 for C57BL/6 + HS; 1.14 ± 0.27 for Fat-1 and 0.79 ± 0.11 for Fat-1 + HS (Table 2). 

### 3.5. Analysis of Strength, Contractile Properties and Resistance to Fatigue in the Soleus Muscle

**Figure 1:**
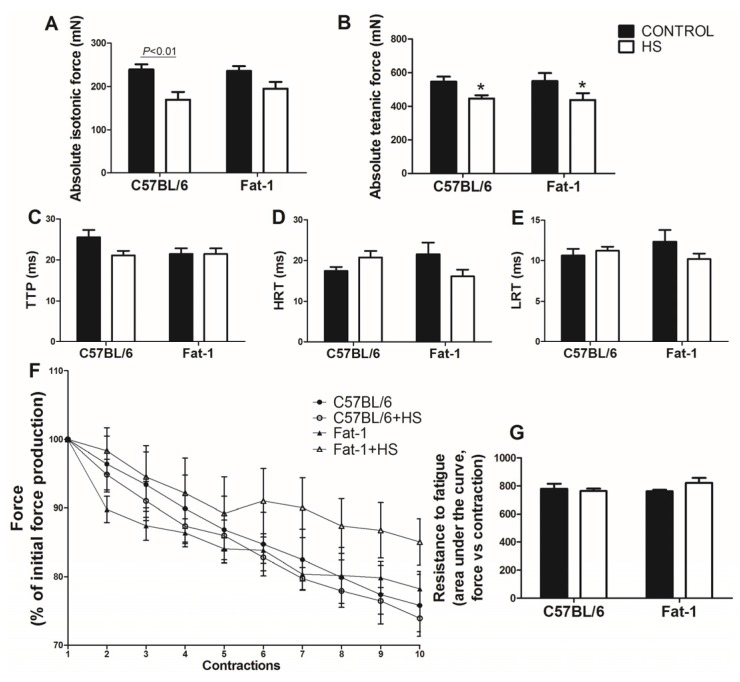
Muscle strength, contractile properties and muscle fatigue of the four groups studied (C57BL/6, C57BL/6 + HS, Fat-1, Fat-1 + HS) in the soleus muscle. Muscle strength: (**A**) Absolute isotonic force. (**B**) Absolute tetanic force. Contractile properties: (**C**) TTP; (**D**) HRT; (**E**) LRT. Muscle fatigue: (**F**) and (**G**) Resistance to fatigue (force vs contraction). Values are presented as mean ± SEM, *n* = 5–8 animals. The results were compared using two-way ANOVA and Bonferroni post-hoc test. In (**A**), *p* < 0.01 indicates significant difference using the Bonferroni *post-hoc* test. In (**B**), * *p* < 0.01 for C57BL/6 and Fat-1 groups vs. C57BL/6 + HS and Fat-1 + HS groups (main effect of HS), using two-way ANOVA only (no statistical differences using the Bonferroni *post-hoc* test). HS: hindlimb suspension; TTP: time to peak; HRT: half relaxation time; LRT: late relaxation time.

Absolute isotonic force was decreased in the soleus muscle due to HS in the C57BL/6 mice (*p <* 0.01). This decrease was less pronounced in balanced diet-fed Fat-1 mice (Figure 1A). Absolute tetanic force in the soleus muscle was not significantly different between C57BL/6 and Fat-1 mice, as indicated by the intra-group analysis using Bonferroni *post-hoc* test. However, as indicated by ANOVA (with no *post-hoc* test difference), HS reduced the absolute tetanic force in the soleus muscle (*p <* 0.01) as compared with non-HS in both groups (Figure 1B). The contractile properties (TTP, HRT, LRT in Figure 1C–E, respectively) as well as the resistance to fatigue (Figure 1F,G) did not change significantly in the soleus muscle among the groups. Based on the inclination of the fatigue curve, there is a trend for increase fatigue resistance mainly after 5th contraction in Fat-1 mice when compared with C57BL/6 wild type. The values were: −1.13 ± 0.21 for C57BL/6; −1.04 ± 0.24 for C57BL/6 + HS; −0.44 ± 0.35 for Fat-1; and −0.65 ± 0.28 for Fat-1 + HS.

### 3.6. CSA of the Soleus Muscle and of Soleus Muscle Fibers

**Figure 2:**
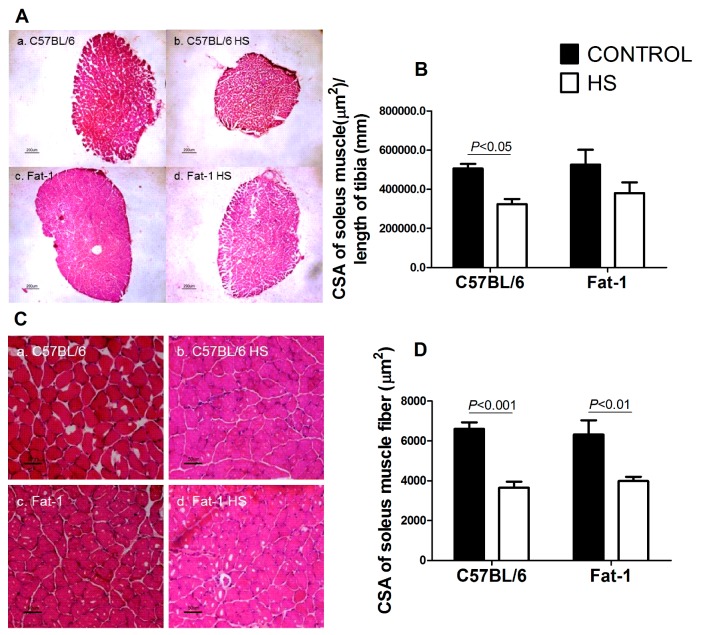
Cross-sectional areas (CSA) of the soleus muscle and soleus muscle fibers of the four groups studied (C57BL/6, C57BL/6 + HS, Fat-1, Fat-1 + HS). (**A**) Representative histological hematoxylin and eosin staining images of cross-sectional areas of the whole soleus muscle. a. C57BL/6; b. C57BL/6 + HS; c. Fat-1; d. Fat-1 + HS. Reference bar represents 200 µm. (**B**) Cross-sectional area of the soleus muscle. Values are presented as mean ± SEM, *n* = 7–8 animals. (**C**) Representative histological hematoxylin and eosin staining images of cross-sectional areas of soleus muscle fibers. a. C57BL/6; b. C57BL/6 + HS; c. Fat-1; d. Fat-1 + HS. Reference bar represents 50 µm. (**D**) Cross-sectional areas of soleus muscle fibers. Values are presented as mean ± SEM, *n* = 6–8. The results were compared using two-way ANOVA and Bonferroni *post-hoc* test. In (**B**) and (**D**), *p* values indicate significant differences using the Bonferroni *post-hoc* test. HS: hindlimb suspension; CSA: Cross-sectional area. 3.7. Activities of Cathepsin L and 26S Proteasome in the Gastrocnemius Muscle, and Contents of Atrogin-1/MAFbx and MuRF1 in the Soleus Muscle.

Soleus muscle CSA was significantly decreased (*p <* 0.05) in the C57BL/6 mice due to HS. The decrease in soleus muscle CSA induced by HS was less pronounced in the balanced diet-fed Fat-1 mice (Figure 2A,B). HS decreased CSA of the soleus muscle fibers in both groups (C57BL/6 vs. C567BL/6 + HS, *p <* 0.001 and Fat-1 vs. Fat-1 + HS, *p <* 0.01) (Figure 2C,D). 

**Figure 3:**
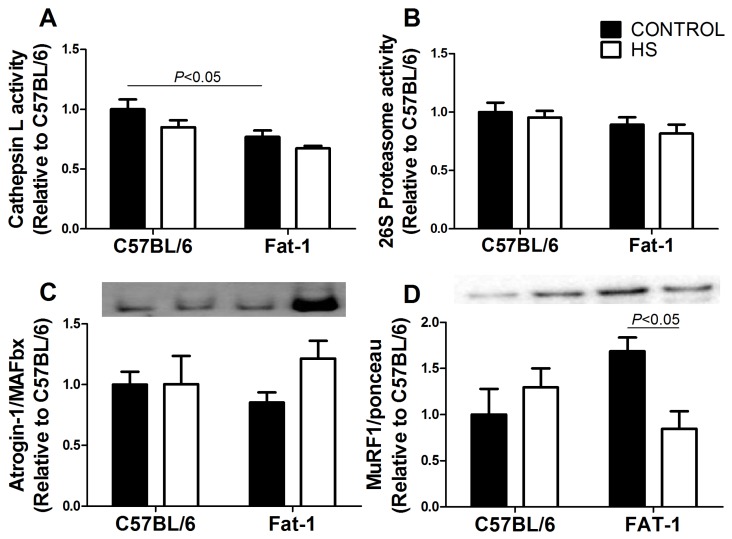
(**A**) Cathepsin L activity in the gastrocnemius muscle of the four groups studied (C57BL/6, C57BL/6 + HS, Fat-1, Fat-1 + HS). (**B**) 26S proteasome activity in the gastrocnemius muscle. (**C**) atrogin-1/MAFbx and (**D**) MuRF-1 content in soleus muscle. Values are presented as mean ± SEM on the basis of total protein loading as indicated by the Ponceau S measurement (**C**,**D**) and expressed relative to the C57BL/6 control group, *n* = 5–8 animals. The results were compared using two-way ANOVA and Bonferroni *post-hoc* test. In (**A**) and (**D**), *p* < 0.05 indicates significant differences using the Bonferroni post-hoc test. HS: hindlimb suspension.

Cathepsin L activity was lowered (*p <* 0.05) in the gastrocnemius muscle of Fat-1 as compared with the C57BL/6 mice (Figure 3A). HS had no significant effect on cathepsin L in both groups. The activity of 26S proteasome was not changed in the gastrocnemius muscle after two-week HS in both groups (Figure 3B). The content of atrogin-1/MAFbx was not significantly changed (Figure 3C) but MuRF1 content was decreased *(p <* 0.05) in the soleus muscle due to HS in Fat-1 mice (Figure 3D). C57BL/6 mice did not exhibit significant changes due to HS in atrogin-1/MAFbx and MuRF-1 contents. 

### 3.7. Protein Synthesis-Associated Signaling in Soleus Muscle

Protein synthesis signaling had a slight reduction (without significant changes as indicated by ANOVA and the Bonferroni *post-hoc* test) due to HS: p-Akt, p-S6, total S6, total GSK3B, phospho- and total-ERK 1/2 (Figures S2 and S3). p-GSK3B/total GSK3B ratio was increased (*p <* 0.01) in the Fat-1 group due to HS as indicated by the Bonferroni *post-hoc* test (Figure S2L). 

## 4. Discussion

This study is the first to use Fat-1 transgenic mice in an experimental model of skeletal muscle disuse atrophy in which neural activity was maintained [25,26]. We were able to characterize the soleus muscle disuse atrophy in an experimental model that mimics a bed rest condition in humans [47]. The measurements were performed after 14 days of HS in order to investigate the changes in the period when muscles composed mainly of oxidative/slow twitch/type I fibers (in soleus and gastrocnemius muscles) have the highest percentage of mass loss [2]. Novel results are reported that motivate the investigation of the full mechanisms involved.

Balanced diet-fed Fat-1 mice, with no HS, had decreased total ω-6 fatty acid content and ω-6/ω-3 fatty acids ratio in the gastrocnemius skeletal muscle associated with higher EDL wet muscle mass, when compared with the C57BL/6 mice. Fat-1 mice had also decreased cathepsin L activity in the gastrocnemius muscle and increased soleus muscle total protein content. Fat-1 mice had a lowered dry mass loss and partially preserved the absolute isotonic force and CSA in the soleus muscle after HS period. 

HS reduced the body weight and fat mass in both groups as also previously reported by others [48,49]. We observed a decrease of the weight gain in the animals submitted to HS. Similar results were reported by Lloyd et al. (2014) in C57BL/6 mice after 7, 14 and 21 days of HS [49]. We also observed a reduction in the fat mass that is in accordance with reports by others [50,51,52].

As reported in our last work [1] and by others [2,53,54,55,56,57], the percentages of skeletal muscle mass loss induced by HS were different among the skeletal muscles. Soleus muscle was the most affected. Soleus muscle has predominantly oxidative/slow twitch/type I fibers that are associated with greater mass loss due to gravity withdrawn conditions as in HS [2]. The HS effects were less pronounced in muscles with a predominance of glycolytic/fast twitch/type II fibers. Hanson et al. (2013) reported a 25% reduction of soleus muscle mass in C57BL/6 mice after 14 days of HS, equal to the dry weight decrease herein described (25%) [58]. Attenuation in the decrease of soleus muscle dry mass induced by HS was observed in Fat-1 mice. 

The gastrocnemius muscle was the second mostly affected (in the percentage of mass loss due to HS). Gastrocnemius muscle of Fat-1 mice had an increase of ω-3 PUFAs, decrease of ω-6 PUFAs, and consequently a decrease in the ω-6/ω-3 PUFA ratio [24,27]. The effects of the EPA are more pronounced as compared with DHA in conditions of muscle disuse atrophy [1] or aging [59]. 

HS had very little effect on skeletal muscle fatty acid composition. However, we measured the fatty acid composition of the gastrocnemius muscle only, so we cannot rule out the possibility that differences in composition of fatty acids [60] and in intramyocellular lipid droplet accumulation [61] may occur between slow- and fast-twitch muscle fibers and whether this may have a role in the muscle-specific effects reported. Rat slow-twitch fibers have a lower content of EPA and DHA and also a higher ω-6/ω-3 PUFA ratio [60]. It may be associated to the increased susceptibility of the soleus muscle to HS-induced atrophy. The increased content of ω-3 PUFA in Fat-1 mice may be more relevant to the soleus muscle that exhibited the greatest atrophy attenuation.

HS decreased skeletal muscle force and resistance to fatigue as also reported by others [58,62,63]. Fat-1 mice submitted to HS had lower decrease of the absolute isotonic force and a less pronounced reduction in skeletal muscle force after the fifth contraction (without significant changes) and it became more pronounced by HS. ω-3 PUFAs preserve skeletal muscle function in humans with skeletal muscle atrophy [16,17,64] and this effect is associated with increased mitochondrial biogenesis [17,59], improved ATP resynthesis and/or sarco/endoplasmic reticulum Ca^2+^-ATPase (SERCA) pump activity during skeletal muscle contraction [65]. The same reduction in mitochondrial biogenesis is reported in mice during hindlimb suspension [54]. The ω-3 PUFAs may have a role in preventing the decrease in mitochondria biogenesis, which contributes, not only to improve fatigue resistance and contractile properties but for the maintenance of muscle mass during HS suspension.

Peoples and McLennan (2010) investigated the effects of an eight-week administration of a diet rich with in saturated fatty acids, ω-6 or ω-3 PUFAs in rats. Animals fed with a diet rich in ω-3 PUFAs (high-DHA tuna fish oil) had a delay in muscle fatigue, improved contractile function recovery, and reduced consumption of oxygen during skeletal muscle contraction. The protocol of electrical stimulation used to promote skeletal muscle fatigue was different from the one herein used; 1 Hz vs. 100 Hz of frequency and gastrocnemius-plantaris-soleus vs. soleus muscles, respectively [66]. The same group, using a slightly different electrical stimulation protocol (5 Hz), reported that, after 15 weeks of low or moderate fish oil (0.31% and 1.25%, respectively) containing diet (high-DHA tuna fish oil), no differences were observed in the maximum skeletal muscle force when compared with control animals (10% of olive oil according to body weight). The resistance to fatigue was elevated in the fish oil-fed group and this observation was associated with increased incorporation of DHA in sarcoplasmic membranes. ω-3 PUFAs may then improve fatigue resistance in a muscle type-specific way, as it had an effect in glycolytic/white gastrocnemius muscle but not in oxidative/soleus muscle [67]. In this study no increased tetanic force was reported. 

The attenuation of the CSA reduction due to HS in the soleus muscle of Fat-1 mice is in line with the findings reported for the muscle dry mass and absolute isotonic force, in which the statistical differences between the Fat-1 and Fat-1 + HS groups are abolished. HS did not change the levels of the protein degradation signaling in soleus muscle. Two weeks of HS did not increase the cathepsin L activity as also reported in our previous study in rats [1], neither 26S proteasome activity nor atrogin-1/MAFbx content. The protein degradation activity may play a significant role at the beginning of the process, contributing to the establishment of the skeletal muscle atrophy. This metabolic feature does not persist in a prolonged period of muscle disuse induced atrophy as reported by others [2,3,49,58,68]. 

The levels of some signaling molecules (Akt, S6, ERK 1/2) of the protein synthesis pathway were slightly (not significantly) decreased due to HS, as also reported by others after muscle disuse atrophy [2,3,49,58,68]. The ω-3 PUFAs promote anabolism by increasing insulin sensitivity and activating the Akt-mTOR-S6 signaling [20]. The ω-3 PUFAs improve mitochondrial biogenesis [17,59] and modify mitochondrion membrane composition [65]. 

Lim et al. (2013) investigated skeletal muscle atrophy in Fat-1 mice using an acute SCI model. After 28 days, the Fat-1 mice exhibited a reduced impact of the SCI and improved locomotor recovery as compared with the C57BL/6 wild-type mice [25]. The same group described that, after seven days of peripheral nerve injury (PNI) (sciatic nerve), Fat-1 mice displayed neuroprotective effects and had a pro-regenerative potential increased as compared with C57BL/6 wild-type [26]. Both studies were performed on a diet rich in ω-6 PUFAs and poor in ω-3 PUFAs. 

We previously reported that supplementation with high levels of ω-3 PUFAs, which induced a four-fold decrease in gastrocnemius ω-6/ω-3 PUFA ratio, did not attenuate the soleus muscle mass loss after 14 days of HS [1]. In the present study, a chronic increase in the amount of ω-3 PUFAs, with a ω-6/ω-3 PUFA ratio only 56% lower than that of the control, preserved the skeletal muscle mass in HS with minor changes in protein synthesis and degradation signaling. 

The HS effects on soleus muscle atrophy were possibly less pronounced in Fat-1 mice due to PUFAs anabolic actions. The muscle contraction preserves muscle mass but, without the mechanical loading, it is insufficient to prevent the complete muscle mass loss. The effects of ω-3 PUFAs are possibly increased when associated with muscle activation/loading conditions as in physical exercise, neuromuscular electrical stimulation or mechanical loading. 

Despite the originality and relevance of the present study, some limitations have to be considered. The authors did not investigate the effect of a ω-6 PUFAs rich diet, the early consequences of HS in both groups (for instance three or five days of HS) or upstream degradation markers, downstream effectors and final responses on protein synthesis and degradation. Caspase/calpain activity production of reactive oxygen species (ROS) and endoplasmic reticulum stress may also be involved in the HS-induced muscle atrophy [9,69,70,71].

## 5. Conclusions

In conclusion, a slight increase of ω-3 PUFAs in the skeletal muscle tissue preserved skeletal muscle mass when the mice faced a muscle disuse condition. The consequences (decrease of skeletal muscle mass, absolute isotonic force and soleus muscle CSA) induced by the skeletal muscle disuse were less pronounced in balanced diet-fed Fat-1 mice.

## Figures and Tables

**Table 1:** Body weight, skeletal muscles and fat depots mass and soleus protein content of the four groups: C57BL/6, C57BL/6 + HS, Fat-1, and Fat-1 + HS.

	C57BL/6	C57BL/6 + HS	Fat-1	Fat-1 + HS
**Body weight**				
Initial body weight (g)	23 ± 0.6	23 ± 0.5	22 ± 0.4	24 ± 0.7
Increase or decrease of body mass after 14 days (g)	1.6 ± 0.3	−0.7 ± 0.3 ^b^	1.3 ± 0.4	−1.7 ± 0.8 ^c^
**Soleus muscle**				
Wet weight (mg/mm tibia length)	0.456 ± 0.019	0.263 ± 0.010 ^c^	0.435 ± 0.013	0.319 ± 0.021 ^c,x^
Percent loss due to HS in wet weight		42%		27%
Dry weight (mg/mm tibia length)	0.149 ± 0.010	0.112 ± 0.010 ^a^	0.151 ± 0.017	0.137 ± 0.012
Percent loss due to HS in dry weight		25%		9%
**Gastrocnemius muscle**				
Wet weight (mg/mm tibia length)	6.945 ± 0.168	5.334 ± 0.144 ^c^	6.608 ± 0.213	5.473 ± 0.188 ^c^
Percent loss due to HS in wet weight		23%		17%
Dry weight (mg/mm tibia length)	1.683 ± 0.046	1.320 ± 0.046 ^c^	1.649 ± 0.056	1.355 ± 0.063 ^b^
Percent loss due to HS in dry weight		22%		18%
**Plantaris muscle**				
Wet weight (mg/mm tibia length)	0.892 ± 0.019	0.697 ± 0.010 ^c^	0.914 ± 0.013	0.784 ± 0.021 ^c,y^
Percent loss due to HS in wet weight		22%		14%
Dry weight (mg/mm tibia length)	0.218 ± 0.009	0.171 ± 0.006 ^b^	0.232 ± 0.012	0.187 ± 0.010 ^b^
Percent loss due to HS in dry weight		22%		19%
**Tibialis anterior muscle**				
Wet weight (mg/mm tibia length)	2.237 ± 0.074	1.860 ± 0.063 ^c^	2.369 ± 0.098	1.949 ± 0.071 ^b^
Percent loss due to HS in wet weight		17%		18%
Dry weight (mg/mm tibia length)	0.635 ± 0.015	0.565 ± 0.025	0.645 ± 0.034	0.536 ± 0.025 ^a^
Percent loss due to HS in dry weight		11%		17%
**EDL muscle**				
Wet weight (mg/mm tibia length)	0.478 ± 0.020	0.434 ± 0.021	0.544 ± 0.015 ^x^	0.469 ± 0.017 ^a^
Percent loss due to HS in wet weight		9%		14%
Dry weight (mg/mm tibia length)	0.141 ± 0.009	0.122 ± 0.009	0.137 ± 0.007	0.128 ± 0.008
Percent loss due to HS in dry weight		14%		7%
**Subcutaneous fat mass**				
Wet weight (mg/cm L)	22.49 ± 2.40	22.55 ± 2.98	21.03 ± 1.30	18.51 ± 2.46
Percent loss due to HS in wet weight		0%		12%
**Epididymal fat mass**				
Wet weight (mg/cm L)	26.23 ± 1.56	15.37 ± 0.81 ^c^	19.82 ± 1.30 ^y^	11.16 ± 1.57 ^c^
Percent loss due to HS in wet weight		41%		44%
**Retroperitoneal fat mass**				
Wet weight (mg/cm L)	6.80 ± 0.93	7.40 ± 0.72	6.72 ± 0.46	4.56 ± 0.54 ^x^
Percent loss due to HS in wet weight		0%		32%
**Mesenteric fat mass**				
Wet weight (mg/cm L)	17.21 ± 2.96	13.35 ± 2.55	15.48 ± 2.57	12.90 ± 3.29
Percent loss due to HS in wet weight		22%		17%
**Soleus total protein content**				
mg/g fresh tissue	34.33 ± 7.84	43.00 ± 9.72	62.47 ± 7.16 ^x^	59.67 ± 1.22

Values are presented as mean ± SEM of at least seven animals. The results were compared using two-way ANOVA and Bonferroni *post-hoc* test. The percentages indicate the effect of HS in the respective groups. ^a^ *p <* 0.05; ^b^ *p <* 0.01; ^c^ *p <* 0.001 for significant differences using the Bonferroni *post-hoc* test between the HS groups and the respective controls. ^x^ *p <* 0.05; ^y^ *p <* 0.01; ^z^ *p <* 0.001 for significant differences using the Bonferroni *post-hoc* test between the C57BL/6 vs. Fat-1 groups (C57BL/6 vs. Fat-1 or C57BL/6 + HS vs. Fat-1 + HS). HS: the hindlimb suspension group; EDL: extensor digitorum longus; L: length of the animal; SEM: standard error of the mean.

**Table 2:** Composition of fatty acids in the gastrocnemius muscle (g/100 g wet weight).

Fatty Acid	Name	C57BL/6	C57BL/6 + HS	Fat-1	Fat-1 + HS
14:0	Myristic	0.01 ± 0.00	0.01 ± 0.00	0.01 ± 0.00	0.01 ± 0.00
16:0	Palmitic	0.29 ± 0.04	0.28 ± 0.02	0.23 ± 0.10	0.30 ± 0.03
16:1 (ω-7)	Hexadecenoic	0.03 ± 0.01	0.04 ± 0.01	0.02 ± 0.01	0.05 ± 0.01 ^c^
17:0	Margaric	-	-	-	-
18:0	Stearic	0.14 ± 0.02	0.13 ± 0.01	0.11 ± 0.05	0.13 ± 0.01
18:1 (ω-9)	Oleic	0.18 ± 0.06	0.15 ± 0.04	0.13 ± 0.07	0.18 ± 0.04
18:1 (ω-11)	Vaccenic	0.04 ± 0.01	0.04 ± 0.01	0.03 ± 0.01	0.04 ± 0.00
18:2 (ω-6)	Linoleic	0.25 ± 0.07	0.21 ± 0.03	0.21 ± 0.09	0.24 ± 0.04
20:0	Eicosanoic	-	-	-	-
20:1 (ω-9)	Eicosenoic	-	-	-	-
18:3 (ω-6)	γ-Linolenic	-	-	-	-
18:3 (ω-3)	α-Linolenic	0.01 ± 0.01	0.01 ± 0.00	0.01 ± 0.01	0.02 ± 0.01
22:0	Docosanoic	-	-	-	-
20:2 (ω-6)	Eicosadienoic	0.01 ± 0.00	-	-	-
20:3 (ω-9)	Eicosatrienoic	0.01 ± 0.00	0.01 ± 0.00	-	-
20:4 (ω-6)	Arachidonic	0.13 ± 0.01	0.13 ± 0.02	0.02 ± 0.01 ^z^	0.01 ± 0.00 ^z^
22:2 (ω-6)	Docosadienoic	-	-	-	—
20:5 (ω-3)	Eicosapentaenoic	-	-	0.01 ± 0.00	0.02 ± 0.01
22:4 (ω-3)	Docosatetraenoic	0.02 ± 0.00	0.02 ± 0.00	-	-
22:5 (ω-6)	Docosapentaenoic	0.02 ± 0.00	0.02 ± 0.00	-	-
22:5 (ω-3)	Docosapentaenoic	0.02 ± 0.00	0.03 ± 0.00	0.05 ± 0.02 ^z^	0.06 ± 0.01 ^z^
22:6 (ω-3)	Docosahexaenoic	0.13 ± 0.02	0.15 ± 0.04	0.15 ± 0.07	0.21 ± 0.03
Total	Saturated	0.44 ± 0.06	0.42 ± 0.03	0.35 ± 0.16	0.44 ± 0.04
	Monounsaturated	0.25 ± 0.08	0.23 ± 0.06	0.18 ± 0.09	0.27 ± 0.06
	Polyunsaturated	0.59 ± 0.11	0.57 ± 0.08	0.45 ± 0.20	0.56 ± 0.06
	ω-3	0.16 ± 0.03	0.19 ± 0.04	0.22 ± 0.10	0.31 ± 0.04 ^a,y^
	ω-6	0.42 ± 0.08	0.39 ± 0.04	0.23 ± 0.10 ^z^	0.25 ± 0.04 ^y^
ω-6/ω-3 ratio		2.62±0.39	2.11 ± 0.27 ^b^	1.14 ± 0.27 ^z^	0.79 ± 0.11 ^y^
Total fat %		1.37±0.25	1.32 ± 0.17	1.25 ± 0.10	1.37 ± 0.16

The determination of fatty acid by gas chromatography was calculated from the tridecanoate triglyceride, which was used as internal standard. Values are presented as mean ± SD, *n* = 6–7 per group. The results were compared using two-way ANOVA and Bonferroni *post-hoc* test. ^a^ *p <* 0.05; ^b^ *p <* 0.01; ^c^ *p <* 0.001 for: significant differences using the Bonferroni *post-hoc* test between the hindlimb suspension groups and the respective controls. ^x^ *p <* 0.05; ^y^ *p <* 0.01; ^z^ *p <* 0.001 for: significant differences using the Bonferroni *post-hoc* test between the C57BL/6 vs. Fat-1 groups (C57BL/6 vs. Fat-1 or C57BL/6 + HS vs. Fat-1 + HS). HS: hindlimb suspension; SD: standard deviation; -: not detected.

## Supplementary Materials

The following are available online at http://www.mdpi.com/2072-6643/9/10/1100/s1, Figure S1: Image to confirm C57BL/6 wild-type mice or Fat-1 transgenic mice lineage; Ponceau S staining used as loading control, and Images of the western blot assays used in this study, Figure S2: Contents of proteins associated with signaling pathways of protein synthesis in the soleus muscle of the four groups studied (C57BL/6, C57BL/6 + HS, Fat-1, Fat-1 + HS), Figure S3: Results of the quantitative analysis of western blot assay used in the Figure S2 of this study.
