# Supplementary material for: Balanced Diet-Fed Fat-1 Transgenic Mice Exhibit Lower Hindlimb Suspension-Induced Soleus Muscle Atrophy
Source: Nutrients. 2017 Oct 6;9(10):1100. doi: 10.3390/nu9101100 (PMC5691716; doi:10.3390/nu9101100)
Supplement: Supplementary file 1 [file nutrients-09-01100-s001.docx]

**Supplementary Materials:**


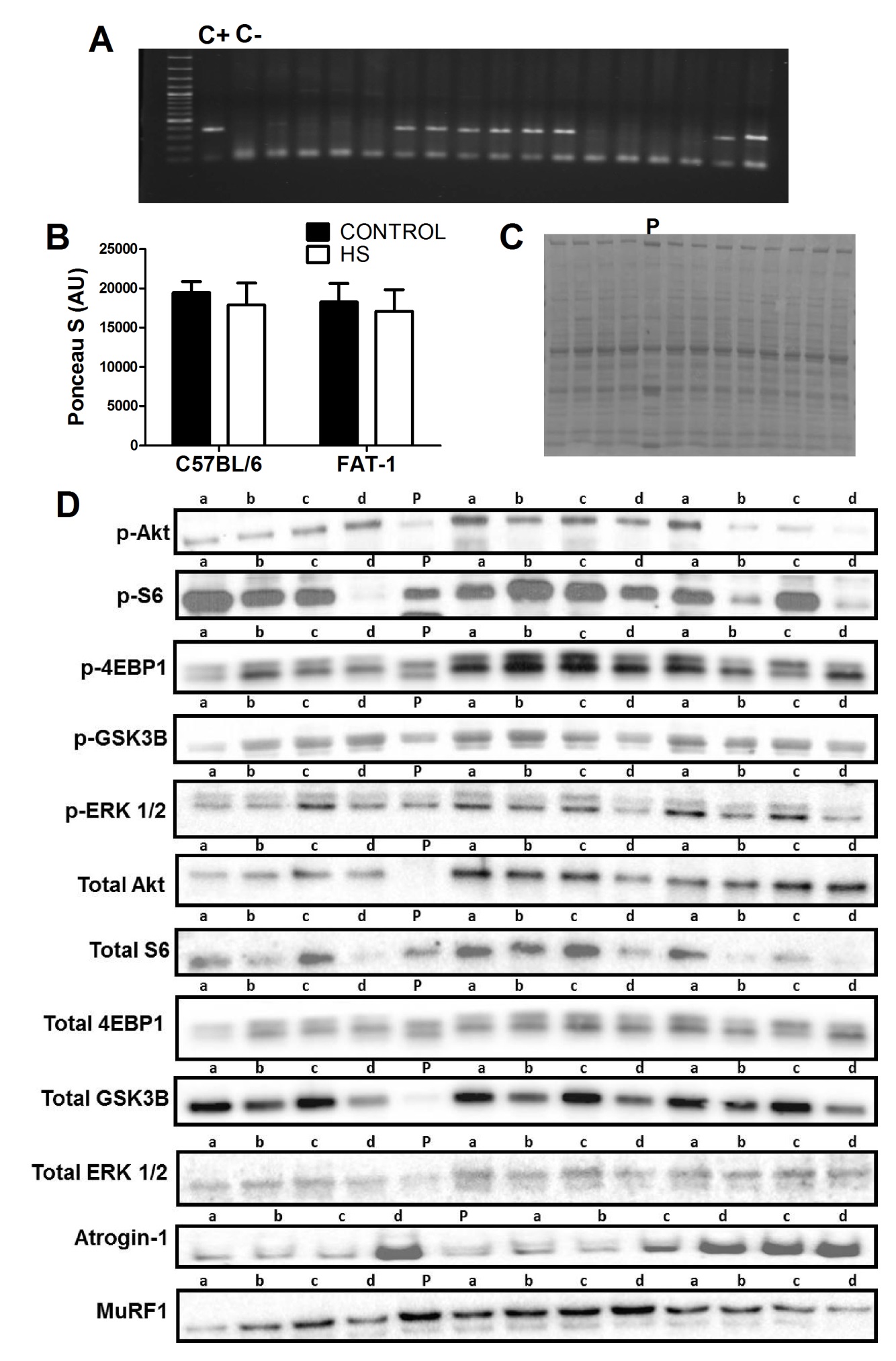


**Figure S1.** Image to confirm C57BL/6 wild-type mice or Fat-1 transgenic mice lineage; Ponceau S staining used as loading control, and Images of the western blot assays used in this study. **A.** Image of agarose at gel 3% after PCR in 21 day-old mice to confirm C57BL/6 wild-type mice or Fat-1 transgenic mice lineage. **B.** Average quantitative analysis of Ponceau S staining. No significant differences were observed. The results were compared using two-way ANOVA and Bonferroni *post-hoc* test, without any differences **C.** Image of the western blot membrane stained with Ponceau S used in this study. **D.** Images used for quantitative analysis of the western blot assays used in this study. **C+:** positive control for Fat-1 transgenic mice; **C-:** negative control for Fat-1 transgenic mice; **P:** pool containing a mixture of equal parts of all samples – used to normalize Ponceau S quantitative results; **HS:** hindlimb suspension; **AU:** arbitrary units; **a:** C57BL/6 wild-type; **b:** C57BL/6 wild-type submitted to hindlimb suspension; **c:** Fat-1 mouse; **d:** Fat-1 submitted to hindlimb suspension mouse.

**
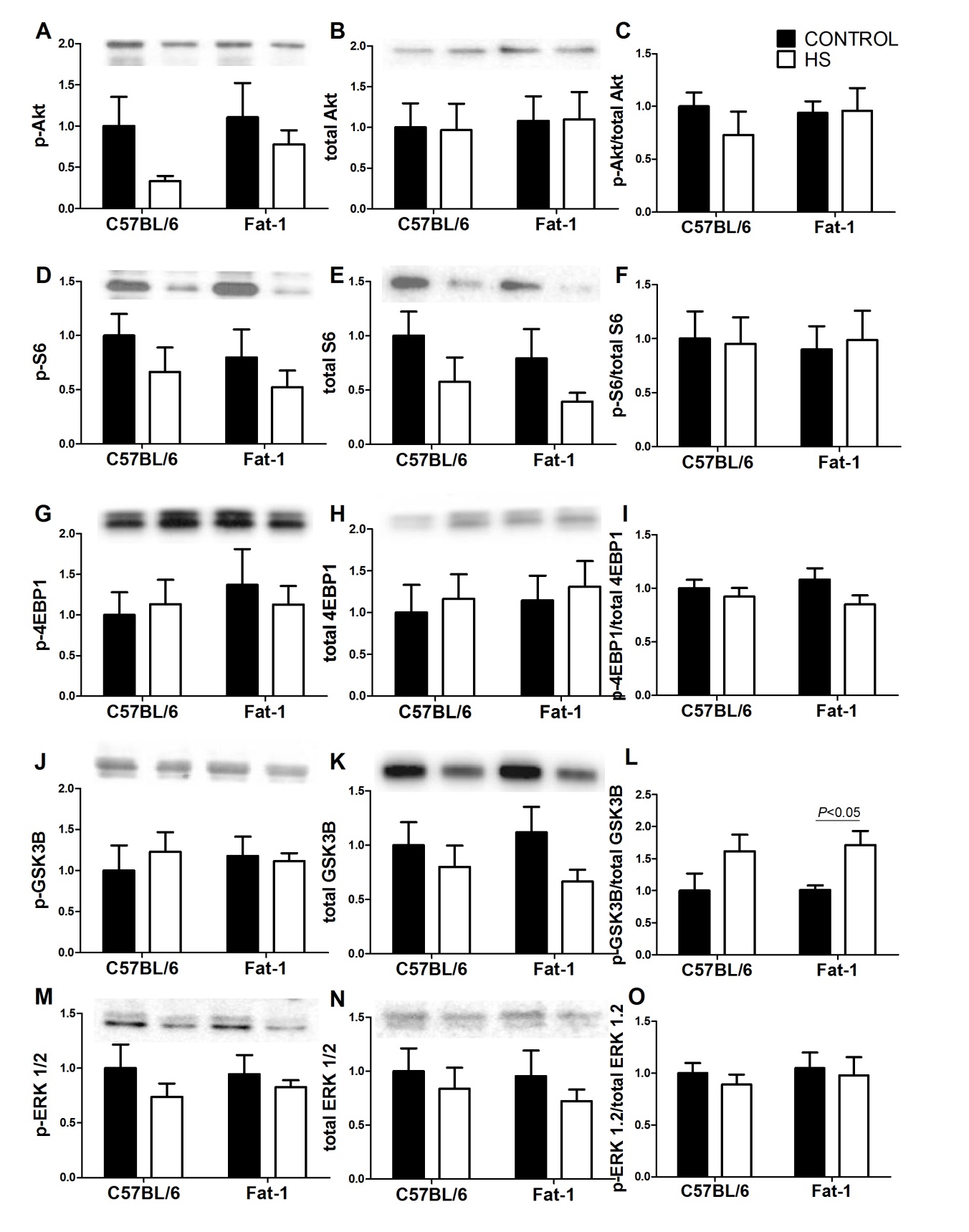
**

**Figure S2.** Contents of proteins associated with signaling pathways of protein synthesis in the soleus muscle of the four groups studied (C57BL/6, C57BL/6+HS, Fat-1, Fat-1+HS): **A.** p-Akt, **B.** total Akt, **C.** p-Akt/Akt total ratio*,* **D.** p-S6, **E.** total S6, **F.** p-S6/S6 total ratio, **G.** p-4EBP1, **H.** total 4EBP1, **I.** p-4EBP1/4EBP1 total ratio, **J.** p-GSK3-beta, **K.** total GSK3-beta, **L.** p-GSK3-beta/total GSK3-beta ratio, **M.** p-ERK 1/2, **N.** total ERK 1/2, **O.** p-ERK 1.2/total ERK 1.2 ratio. Values are presented as mean ± SEM on the basis of total protein loading as indicated by the Ponceau S measurements and expressed relative to the C57BL/6 control group, n=4-6 animals. The results were compared using two-way ANOVA and Bonferroni *post-hoc* test. In **L**, *p* ˂ 0.05 indicates significant differences using the Bonferroni *post-hoc* test**. HS:** hindlimb suspension; **SEM:** standard error of the mean.

**Figure S3.** Results of the quantitative analysis of western blot assay used in the Figure S2 of this study. **A.** p-Akt, **B.** total Akt, **C.** p-Akt/Akt total ratio*,* **D.** p-S6, **E.** total S6, **F.** p-S6/S6 total ratio, **G.** p-4EBP1, **H.** total 4EBP1, **I.** p-4EBP1/4EBP1 total ratio, **J.** p-GSK3-beta, **K.** total GSK3-beta, **L.** p-GSK3-beta/total GSK3-beta ratio. **M.** p-ERK 1/2, **N.** total ERK 1/2, **O.** p-ERK 1.2/total ERK 1.2 ratio. **HS:** hindlimb suspension.
